# Transcription factor IRF5 drives P2X4R^+^-reactive microglia gating neuropathic pain

**DOI:** 10.1038/ncomms4771

**Published:** 2014-05-13

**Authors:** Takahiro Masuda, Shosuke Iwamoto, Ryohei Yoshinaga, Hidetoshi Tozaki-Saitoh, Akira Nishiyama, Tak W. Mak, Tomohiko Tamura, Makoto Tsuda, Kazuhide Inoue

**Affiliations:** 1 Department of Molecular and System Pharmacology, Graduate School of Pharmaceutical Sciences, Kyushu University, 3-1-1 Maidashi, Higashi-ku, Fukuoka 812-8582, Japan; 2 Core Research for Evolution Science and Technology, Japan Science and Technology Agency, Tokyo 102-0076, Japan; 3 Department of Immunology, Yokohama City University Graduate School of Medicine, 3-9 Fukuura, Kanazawa-ku, Yokohama 236-0004, Japan; 4 Princess Margaret Cancer Centre, University Health Network, 620 University Avenue, Toronto, Ontario, Canada M5G 2C1

## Abstract

In response to neuronal injury or disease, microglia adopt distinct reactive phenotypes via the expression of different sets of genes. Spinal microglia expressing the purinergic P2X4 receptor (P2X4R) after peripheral nerve injury (PNI) are implicated in neuropathic pain. Here we show that interferon regulatory factor-5 (IRF5), which is induced in spinal microglia after PNI, is responsible for direct transcriptional control of P2X4R. Upon stimulation of microglia by fibronectin, IRF5 induced *de novo* expression of P2X4R by directly binding to the promoter region of the *P2rx4* gene. Mice lacking *Irf5* did not upregulate spinal P2X4R after PNI, and also exhibited substantial resistance to pain hypersensitivity. Furthermore, we found that expression of IRF5 in microglia is regulated by IRF8. Thus, an IRF8-IRF5 transcriptional axis may contribute to shifting spinal microglia toward a P2X4R-expressing reactive state after PNI. These results may provide a new target for treating neuropathic pain.

## Abstract (web summary)

In response to neuronal injury or disease, microglia adopt distinct reactive phenotypes via the expression of proteins, such as the purinergic P2X4 receptor. Here, Masuda *et al.* show that the transcription factor axis, interferon regulatory factor-8 and -5, drives the expression of P2X4 receptor in microglia and the adoption of a reactive phenotype after peripheral nerve injury.

## Graphical abstract

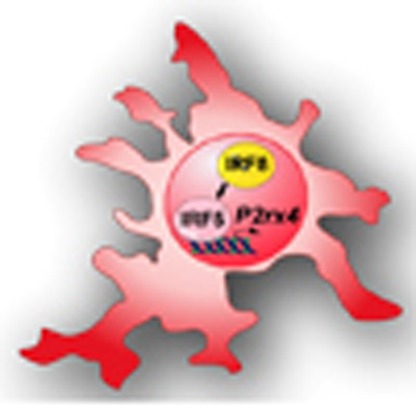

Microglia are the resident immune cells of the central nervous system (CNS). Under physiological conditions, microglia actively move their branched processes to sense pathological alterations or disturbances to ultimately maintain CNS homeostasis^1^,^2^,^3^. Neuronal damage transforms microglia into reactive phenotypes with biochemical alterations, such as the activation of gene transcription^1^,^2^,^4^,^5^. These alterations have been implicated in the disruption of the CNS environment, which is linked to the pathogenesis of various CNS diseases^1^,^4^,^5^. The state of microglial activation is regulated by extracellular signals on surface receptors and also tightly controlled by transcription factors^6^,^7^. We have previously identified interferon regulatory factor-8 (IRF8) as a crucial transcription factor that activates the expression of a variety of genes associated with the activated processes of microglia^8^. However, little is known about the transcriptional regulatory mechanisms underlying the phenotype shift towards a specialized reactive state of microglia.

Injury to the nervous system as a consequence of multiple sclerosis, diabetes mellitus, cancer or traumatic injury often causes a debilitating chronic pain syndrome, termed neuropathic pain^9^,^10^,^11^, which is refractory to currently available treatments^11^. Accumulating evidence indicates the pivotal roles of spinal microglia in neuropathic pain. Following peripheral nerve injury (PNI), microglia in the spinal dorsal horn exhibit an reactive phenotype and upregulated expression of a variety of genes, including purinergic P2X4 receptor (P2X4R)^8^,^12^,^13^. Stimulating these receptors on microglia causes the release of brain-derived neurotrophic factor (BDNF)^14^,^15^, which in turn disinhibits the inhibitory system of the dorsal horn nociceptive network^14^ or enhances excitatory synaptic transmission in the dorsal horn neurons of the spinal cord^16^. The pathological alterations convert innocuous inputs to a nociceptive signal output to the brain, thus contributing to pain hypersensitivity^13^. Therefore, a specific phenotype of spinal microglia characterized by high expression of P2X4R (P2X4R^+^ microglia) after PNI is crucial for the pathogenesis of neuropathic pain^10^,^13^,^15^. However, the molecular machinery that underlies the formation of P2X4R^+^ microglia remains poorly understood.

Here we identify an interferon regulatory factor-5 (IRF5) as a microglial gene whose expression is induced by PNI. IRF5 induces *de novo* expression of P2X4R in activated microglia by fibronectin through a direct binding to the promoter region of the *P2rx4* gene. IRF5-deficient mice show no upregulation of spinal P2X4R after PNI and substantial resistance to pain hypersensitivity. IRF5 is directly regulated by IRF8 in microglia. Thus, our findings indicate that an IRF8–IRF5 transcriptional axis is a critical regulator for shifting towards a P2X4R^+^-reactive phenotype that gates neuropathic pain after PNI and may provide a new target for treating neuropathic pain.

## Results

### PNI induces expression of IRF5 in spinal microglia

To uncover transcription factors that control reactive states of microglia, we initially performed a genome-wide microarray analysis from the spinal cord of wild-type (WT) mice with PNI, which exhibits injured nerve projections and activated microglia, and sorted out the transcription factors upregulated. Among genes evaluated in three or four independent analyses, we identified IRF5 (*P<*0.001) and IRF9 (*P=*0.016) as significant transcription factors increased in the spinal cord ipsilateral to nerve injury (Supplementary Fig. 1a). IRF5 showed a more significant rate of expressional change with high reproducibility compared with that of IRF9 (Supplementary Fig. 1a); we therefore focused on IRF5 in the present study. In the periphery, IRF5 plays pivotal roles in the immune system, such as the induction of pro-inflammatory cytokines^17^ and polarization of M1 macrophages^18^. However, the nature of IRF5 in the CNS is entirely unknown.

**Figure 1:**
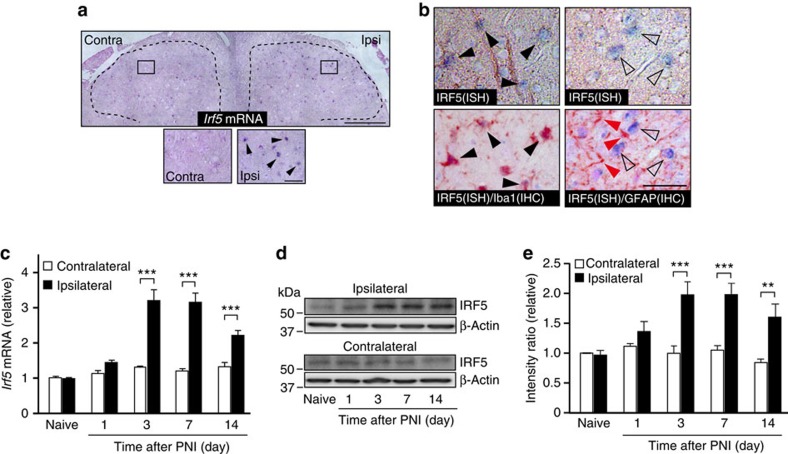
PNI induces the expression of IRF5 in spinal microglia. (**a**) *In situ* hybridization analysis of *Irf5* mRNA in the spinal dorsal horn of mice 7 days after PNI. Black arrowheads indicate *Irf5* mRNA signals. Images are representative of three experiments. Ipsi, ipsilateral; Contra, contralateral. (**b**) Representative images (of three experiments) showing that *Irf5* mRNA signals colocalize with the immunoreactivity of Iba1 (black arrowheads), but not with that of GFAP (red arrowheads). ISH, *in situ* hybridization; IHC, immunohistochemistry. (**c**) Real-time PCR analysis of *Irf5* mRNA in total RNA extracted from spinal cord ipsilateral and contralateral to PNI before (naive) and after PNI. Values represent the relative ratio of *Irf5* mRNA (normalized to the value for *18s* mRNA) to the contralateral side of naive mice (*n=*6; ***P<0.001). (**d**) Representative western immunoblot of IRF5 protein in homogenates from the ipsilateral and contralateral spinal cords of wild-type (WT) mice before (naive) and after PNI. (**e**) Relative band density ratios of IRF5 (normalized to β-actin) to the contralateral side of naive mice at each time point (*n=*6; **P<0.01, ***P<0.001). Values are means±s.e.m. Full-size blots are shown in Supplementary Fig. 8. Scale bar, 200 μm (**a**, upper), 30 μm (**a**, lower), 30 μm (**b**).

To determine the cell types expressing IRF5 in the spinal cord after PNI, we examined the localization of *Irf5* mRNA using *in situ* hybridization combined with immunofluorescence of the cell-type-specific markers. On sections of the fourth lumbar (L4) spinal cord of mice, few or no detectable signals of *Irf5* mRNA were found on the contralateral side. However, PNI increased the signal intensity of *Irf5*, with punctate distribution in the ipsilateral dorsal horn (Fig. 1a, intense violet dots indicated by arrowheads). These signals, which were not observed when hybridized with a corresponding sense probe (Supplementary Fig. 1b), were highly restricted to cells labelled with ionized calcium-binding adapter molecule-1 (Iba1; microglial marker), but not with glial fibrillary acidic protein (GFAP; astrocytic marker) (Fig. 1b). Most of Iba1-positive microglia showed the signals of *Irf5* mRNA in the ipsilateral dorsal horn (42/49 Iba1-positive cells). These results suggest that in the spinal cord, *Irf5* expression is selectively induced in microglia after PNI.

We then determined the temporal expression pattern of *Irf5* mRNA in the spinal cord following PNI via real-time PCR analysis. In support of the *in situ* hybridization results, expression levels of *Irf5* mRNA were low in the spinal cord of naive (that is, before PNI) mice, but markedly increased in the spinal cord ipsilateral to the injury (Fig. 1c). This increase occurred from postoperative day 1, and plateaued by day 3 for >2 weeks (Fig. 1c). In contrast, no detectable change was found in the contralateral spinal cord (Fig. 1c). Western immunoblot analysis using a specific antibody for IRF5 (Supplementary Figs 1c and 13), showed an upregulation of IRF5 protein in the spinal cord after PNI, with a time course and a bilateral difference matching those of *Irf5* mRNA (Fig. 1d,e). Overall, these results indicate that IRF5 is highly restricted to microglia of the dorsal horn, and is dramatically increased after PNI, persisting in the cell for at least 2 weeks.

### Fibronectin-mediated upregulation of P2X4R requires IRF5

**Figure 2:**
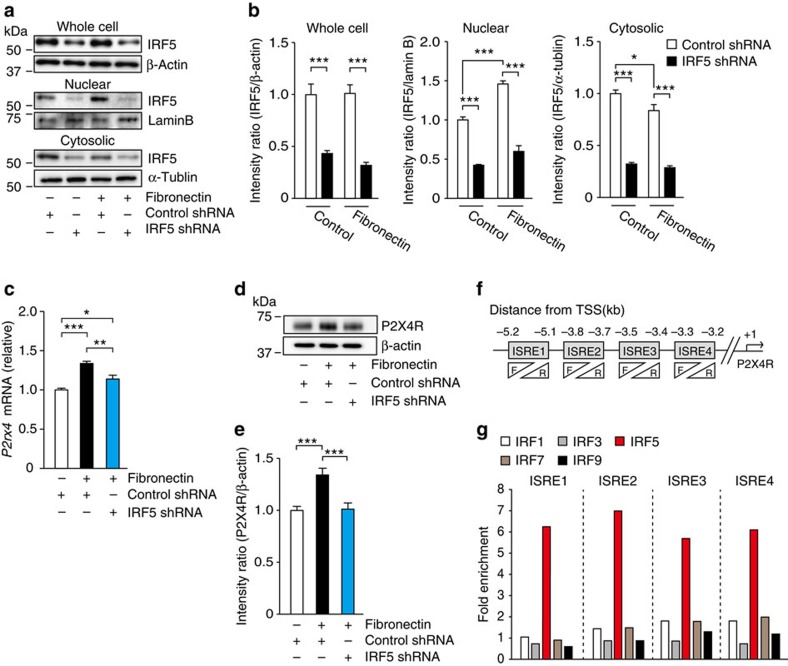
IRF5 is required for fibronectin-mediated upregulation of P2X4R in microglia. (**a**) Representative western immunoblot of IRF5 in whole-cell lysate, and in nuclear or cytosolic components of control or IRF5 shRNA-transduced BV2 cells treated with fibronectin for 4 h. (**b**) Relative band density ratios of IRF5 (normalized to β-actin, lamin B or α-tublin) from control or IRF5 shRNA-transduced BV2 cells treated with fibronectin (*n=*4; *P<0.05, ***P<0.001). (**c**) Real-time PCR analysis of *P2rx4* mRNA in control or *Irf5* shRNA-transduced BV2 cells 6 h after fibronectin treatment. Values represent the relative ratio of *P2rx4* mRNA (normalized to the value for *18s* mRNA) to control shRNA-transduced cells (*n=*6; *P<0.05, **P<0.01, ***P<0.001). (**d**) Representative western immunoblot of P2X4R in control or IRF5 shRNA-transduced BV2 cells 6 h after fibronectin treatment. (**e**) Relative band density ratios of P2X4R (normalized to β-actin) to control shRNA-transduced cells (*n=*6; ***P<0.001). (**f**) Schematic of the four interferon-stimulated response element (ISRE) sites on the promoter region of P2X4R. (**g**) Chromatin immunoprecipitation (ChIP)-qPCR assay of *P2rx4* promoter fragments immunoprecipitated by antibodies for IRF1, IRF3, IRF5, IRF7 or IRF9 in BV2 cells with or without fibronectin, respectively. Values represent the relative ratio of the values of BV2 cells with fibronectin (normalised to the value for normal IgG) to that of cells without fibronectin. Data are representative of three experiments. Values are the mean±s.e.m. Full-size blots are shown in Supplementary Fig. 9.

To explore the function of IRF5 in microglia, we first transduced microglial BV2 cells with a lentiviral vector encoding IRF5, followed by the assessment of the levels of gene transcripts. However, ectopic expression of IRF5 resulted in the minimal effect on an expressional pattern of microglial genes (Supplementary Fig. 2), which is consistent with previous reports noting that full-length IRF5 by itself has a lower potency to drive the reporter gene^19^,^20^. IRF5 is known to predominantly reside in the cytoplasm, and during stimulation-dependent activation, it translocates to the nucleus to activate gene transcription^19^. Thus, to reveal an inherent mechanism that activates microglial IRF5, we focused on several signalling mediators that have been implicated in microglial activation, including toll-like receptor-4 (TLR4)^21^, interferon-gamma (IFN-γ)^22^, adenosine triphosphate (ATP)^23^,^24^ and fibronectin^25^,^26^. These mediators were screened by evaluating their abilities to induce translocation of IRF5. Early reports have shown that IRF5 in macrophages is activated by stimulating TLRs^17^,^20^. In the present study, however, the TLR4 ligand lipopolysaccharide (LPS) unexpectedly did not change the localization of IRF5 in microglia (Supplementary Figs 3 and 14). Likewise, neither ATP nor IFN-γ induced IRF5 translocation to the nucleus (Supplementary Figs 3 and 14). In contrast, IRF5 protein accumulated in the nuclear fraction when cells were stimulated with fibronectin (Fig. 2a,b), and this effect inversely correlated with the reduced level of cytosolic IRF5 (Fig. 2a,b). Total IRF5 protein in whole-cell lysate of microglial cells did not differ before and after treatment with fibronectin (Fig. 2a,b). Therefore, these results indicate that fibronectin activates IRF5 in microglia.

Fibronectin is increased in the spinal cord after PNI^25^ presumably through release from astrocytes^27^ or extravasation from blood flow^28^, and plays crucial roles in *de novo* expression of P2X4R in microglia^25^,^26^,^29^. Therefore, IRF5 may regulate P2X4R expression in microglia. To test this hypothesis, we used BV2 cells in which IRF5 expression had been knocked down by short hairpin RNA (shRNA) targeting IRF5 (Supplementary Fig. 4a,c). We found that IRF5 shRNA markedly suppressed fibronectin-induced upregulation of P2X4R at both the mRNA and protein levels (Fig. 2c–e) without any alteration in the basal expression of P2X4R (Supplementary Fig. 4b), suggesting IRF5-mediated expression of P2X4R in microglia. To determine whether IRF5 directly regulates the transcription of P2X4R, we examined the recruitment of IRF5 to the promoter loci of P2X4R by chromatin immunoprecipitation (ChIP)-qPCR analysis. Using the online software, Genomatix MatInspector ( www.genomatix.de/index.html), we identified four regions that contained an IRF-binding motif, namely interferon-stimulated response element (ISRE), in the promoter of mouse *P2rx4* gene (Fig. 2f). A ChIP-qPCR assay revealed that a fragment of the *P2rx4* promoter containing each putative binding site was amplified with the complex immunoprecipitated with the IRF5 antibody (Fig. 2g). Importantly, the degree of amplification was enhanced by fibronectin treatment (Fig. 2g), implying that activated IRF5 binds to these *p2rx4* promoter regions. In contrast, the amplification rates of each complex immunoprecipitated with antibodies for the other IRF members were not changed by fibronectin treatment (Fig. 2g). Collectively, these findings suggest that IRF5 functions as a transcriptional activator on the *P2rx4* promoter leading to *de novo* expression of P2X4R in microglia (that is, IRF5 shifted microglia towards the P2X4R^+^ phenotype).

### IRF8 directly regulates IRF5 in microglia

**Figure 3:**
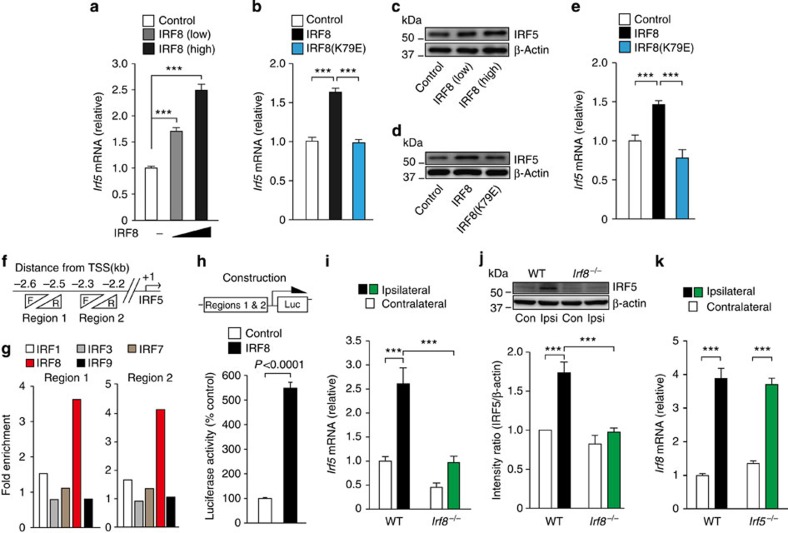
IRF5 expression is directly regulated by IRF8. Real-time PCR analysis of *Irf5* mRNA in BV2 cells transduced with (**a**) IRF8-GFP (high or low dose of IRF8) or GFP alone (control) (*n=*8; ***P<0.001), or (**b**) IRF8-GFP (IRF8), IRF8(K79E)-GFP (IRF8(K79E)) or GFP alone (control) (*n=*8; ***P<0.001). Representative western immunoblot of IRF5 (of three independent experiments) in BV2 cells transduced with (**c**) IRF8-GFP (high or low dose of IRF8) or GFP alone (control), or (**d**) IRF8-GFP (IRF8), IRF8(K79E)-GFP (IRF8(K79E)) or GFP alone (control). (**e**) Real-time PCR analysis of *Irf5* mRNA in primary cultured microglia transduced with IRF8-GFP (IRF8), IRF8(K79E)-GFP (IRF8(K79E)) or GFP alone (control) (*n=*6; ***P<0.001). (**f**) Schematic of the predicted two binding sites of IRF8 on the promoter region of IRF5. (**g**) ChIP-qPCR assay of *Irf5* promoter fragments immunoprecipitated by antibodies for IRF1, IRF3, IRF7, IRF8 or IRF9 in BV2 cells transduced with IRF8-GFP or normal BV2 cells. Values represent the relative ratio of the values of BV2 cells transduced with IRF8-GFP (normalized to the value for normal IgG) to that of normal BV2 cells. Data are representative of three experiments. (**h**) Luciferase activity of BV2 cells transduced with *Irf5* reporter gene, plus IRF8-GFP (IRF8) or GFP alone (control) (*n=*8). (**i**) Real-time PCR analysis of *Irf5* mRNA in the spinal cords of WT and *Irf8*^−/−^ mice 7 days after PNI. Values represent the relative ratio of *Irf5* mRNA (normalized to the value for *18s* mRNA) to the contralateral side of WT mice (*n=*7; ***P<0.001). (**j**) Upper: representative western immunoblots of IRF5 in the spinal cords of WT and *Irf8*^−/−^ mice 7 days after PNI. Lower: relative band density ratios of IRF5 (normalized to β-actin) to the contralateral side of WT mice (*n=*6; ***P<0.001). (**k**) Real-time PCR analysis of *Irf8* mRNA in total RNA extracted from the spinal cords of WT and *Irf5*^−/−^ mice 7 days after PNI. Values represent the relative ratio of *Irf8* mRNA (normalized to the value for *18s* mRNA) to the contralateral side of WT mice (*n=*7; ***P<0.001). Values are the mean±s.e.m. Full-size blots are shown in Supplementary Fig. 10.

We have previously shown that IRF8 in mice spinal cord is markedly upregulated in microglia, and plays a crucial role in their activation^8^. Our results showed that the expression pattern of IRF8 in the spinal cord resembled that of IRF5 (Fig. 1). We therefore examined the hierarchy and interaction between IRF5 and IRF8 in microglia. BV2 cells transduced with green fluorescent protein (GFP)-tagged IRF8 (IRF8-GFP)^8^ significantly increased the expression of IRF5 in a manner that was dependent on the level of expression of IRF8 (Fig. 3a,c). However, the mutant IRF8 in which lysine at amino acid position 79 was replaced with Glu (IRF8(K79E)), resulting in a lack of DNA binding activity^30^, failed to increase IRF5 (Fig. 3b,d). IRF8-mediated upregulation of IRF5 expression was verified using primary cultured microglia transduced with IRF8-GFP (Fig. 3e). We further investigated whether IRF8 directly binds to the promoter loci of IRF5. Previous experiments by genome-wide ChIP coupled to DNA sequencing (ChIP-seq) assay in myeloid progenitor cells revealed two salient IRF8 ChIP-seq peaks localized on the promoter region of IRF5 (ref. 31). We then examined whether IRF8 binds to these sites (regions 1 and 2; Fig. 3f) in microglial cells by ChIP-qPCR analysis, and validated binding of IRF8 to predicted binding sites for IRF5 (Fig. 3g). These binding intensities were much higher in cells transduced with IRF8-GFP compared with normal cells (Fig. 3g). In contrast, binding activities of the other IRF members were indistinguishable between the two types of cells (Fig. 3g). To confirm whether IRF8 transactivates *Irf5* gene expression through these binding sites, we performed a luciferase assay using a reporter containing the two target regions of the mouse *Irf5* gene (Fig. 3h). We found that luciferase activity was approximately five-fold higher in IRF8-transduced cells compared with GFP-transduced cells (Fig. 3h). Together, these findings suggest that IRF5 is directly regulated by IRF8 in microglia.

We then asked whether IRF8 regulates the expression of IRF5 in microglia *in vivo*. We found that PNI-induced upregulation of IRF5 was inhibited in the spinal cord of mice lacking IRF8 (*Irf8*^*−/−*^) both at the mRNA and protein level (Fig. 3i,j), indicating that the expression of IRF5 in spinal microglia is IRF8-dependent. The increase in the expression of spinal IRF8 after PNI was indistinguishable between WT and IRF5-deficient (*Irf5*^*−/−*^) mice (Fig. 3k). Therefore, these results indicate that in spinal microglia, IRF5 is a downstream molecule of and is regulated by IRF8.

### IRF8-mediated *de novo* expression of P2X4R requires IRF5

**Figure 4:**
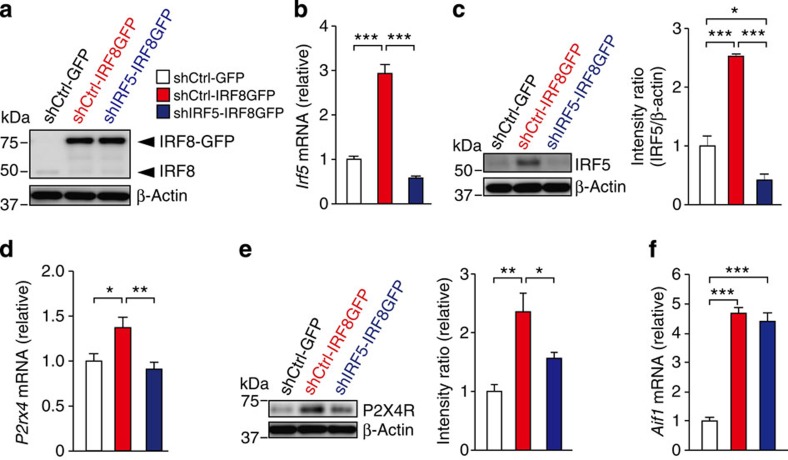
IRF8-mediated P2X4R upregulation in microglia depends on IRF5. Microglial BV2 cells transduced with lentiviral vector encoding IRF5 or control shRNA sequences expressed under a H1 promoter upstream of an EF-1α promoter-IRF8-GFP or GFP expression cassette (shIRF5-IRF8-GFP, shCtrl-IRF8-GFP or shCtrl–GFP). (**a**) Representative western immunoblot of IRF8 (of four experiments) in whole-cell lysates of BV2 cells transduced with each vector. (**b**) Real-time PCR analysis of *Irf5* mRNA in BV2 cells transduced with each vector (*n=*6–8; ***P<0.001). (**c**) Left: representative western immunoblots of IRF5 in BV2 cells transduced with each vector. Right: relative band density ratios of IRF5 (normalized to β-actin) to the shCtrl-GFP-transduced cells (*n=*4; *P<0.05, ***P<0.001). (**d**) Real-time PCR analysis of *P2rx4* mRNA in BV2 cells transduced with each vector (*n=*6–8; *P<0.05, **P<0.01). (**e**) Left: representative western immunoblots of P2X4R in BV2 cells transduced with each vector. Right: relative band density ratios of P2X4R (normalised to β-actin) to the shCtrl-GFP-transduced cells (*n=*4; *P<0.05, **P<0.01). (**f**) Real-time PCR analysis of *Aif1* mRNA in BV2 cells transduced with each vector (*n=*6–8; ***P<0.001). Values are the mean±s.e.m. Full-size blots are shown in Supplementary Fig. 11.

As forced expression of IRF8 in microglia has been shown to upregulate the expression of P2X4R (ref. 8), we hypothesized that IRF8-mediated P2X4R upregulation in microglia may be mediated by IRF5. To test this, we constructed viral vectors encoding IRF5 or control shRNA sequences expressed under an H1 promoter upstream of an EF-1α promoter-IRF8-GFP expression cassette (shIRF5-IRF8-GFP or shCtrl-IRF8-GFP, respectively), and performed gene transfer experiments (Fig. 4a). Cells transduced with shCtrl-IRF8-GFP showed increased expression of P2X4R and IRF5 compared with control cells (shCtrl-GFP) both at the mRNA and protein level (Fig. 4b–e). In contrast, the IRF8-mediated expression of P2X4R was markedly attenuated in cells with IRF5 knockdown (Fig. 4d,e), suggesting that IRF8-mediated *de novo* expression of P2X4R requires IRF5. Meanwhile, expression of *Aif1* (encodes microglial Iba1), which is thought to be directly controlled by IRF8 (ref. 8), was not affected by IRF5 shRNA (Fig. 4f), indicating that knockdown of IRF5 does not result in a global impairment of IRF8-regulated gene expression. Collectively, IRF8-induced IRF5 expression may regulate the expression level of P2X4R in microglia.

### IRF5 is required for upregulation of spinal P2X4R after PNI

**Figure 5:**
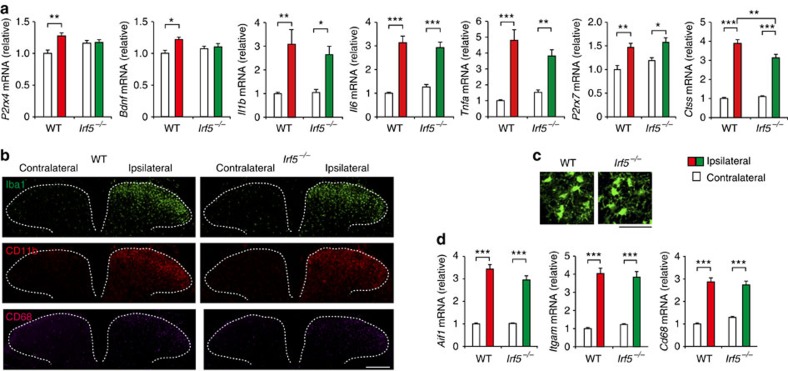
IRF5 is required for *de novo* expression of P2X4R in the spinal cord after PNI but without microglial cellular alterations. (**a**) Real-time PCR analysis of mRNAs of microglial genes in the spinal cords of WT and *Irf5*^−/−^ mice 7 days after PNI. Values represent the relative ratio of mRNA (normalized to the value for *18s* mRNA) to the contralateral side of WT mice (*n=*6, *P<0.05, **P<0.01). Representative images (of three experiments) showing immunofluorescence labelling of (**b**) Iba1 (green), CD11b (red) and CD68 (purple) in the L4 spinal cord of *Irf5*^−/−^ and WT littermates 7 days after PNI (scale bar, 200 μm), or (**c**) Iba1 in the L4 ipsilateral spinal cord of *Irf5*^−/−^ and WT littermates 7 days after PNI (scale bar, 50 μm). (**d**) Real-time PCR analysis of *Aif1*, *Itgam* and *Cd68* mRNA in the spinal cords of WT and *Irf5*^−/−^ mice 7 days after PNI. Values represent the relative ratio of mRNA (normalized to the value for *18s* mRNA) to the contralateral side of WT mice (*n=*6, ***P<0.001). Values are the mean±s.e.m.

We next investigated whether IRF5 was crucial for regulating the expression of P2X4R in microglia after PNI. Consistent with previous studies^8^,^32^, the transcripts of *P2rx4* were significantly increased 7 days after PNI in WT mice (Fig. 5a). In contrast, expression of *P2rx4* was not altered in *Irf5*^*−/−*^ mice (Fig. 5a), emphasizing the importance of IRF5 for the upregulation of P2X4R in spinal microglia. P2X4R-stimulated microglia express and release BDNF, which is a crucial factor for producing tactile allodynia^13^,^14^. Therefore, we determined the expression of BDNF in the spinal cord of *Irf5*^*−/−*^ mice. PNI increased the expression of *Bdnf* transcripts in the spinal cord of WT mice, whereas these changes were not observed in *Irf5*^*−/−*^ mice (Fig. 5a).

IRF5 is reportedly critical for M1-related gene expression, including pro-inflammatory cytokines in macrophages (Supplementary Fig. 5)^17^,^18^. Therefore, we further quantified transcripts of microglial genes related to inflammatory responses (IL-1β (*Il1b*), IL-6 (*Il6*), TNF-α (*Tnfa*), P2X7R (*P2rx7*), and cathepsin S (*Ctss*))^33^,^34^,^35^. Surprisingly, *Irf5*^*−/−*^ mice showed comparable expressions of *Il1b*, *Il6*, *Tnfa* and *P2rx7* to WT mice (Fig. 5a), and a slight inhibition of *Ctss* expression in *Irf5*^*−/−*^ mice was observed (Fig. 5a). Together, IRF5 deficiency specifically downregulates the expression of P2X4R and BDNF at the transcriptional level in spinal microglia after PNI.

### IRF5-independent cellular alterations of microglia after PNI

In response to PNI, spinal microglia undergo morphological hypertrophy and proliferation^8^,^22^. Iba1 staining revealed equivalent morphological alterations of spinal dorsal horn in *Irf5*^*−/−*^ compared with WT mice (Fig. 5b,c). Furthermore, immunofluorescence patterns of CD11b and CD68 (markers of activated microglia) were indistinguishable between the two genotypes (Fig. 5b). These expressional patterns of microglial markers were verified by real-time PCR analysis (Fig. 5d). These results indicate that PNI-induced morphological alterations of microglia in the spinal cord are independent of IRF5.

### Tactile allodynia after PNI is dependent on IRF5

**Figure 6:**
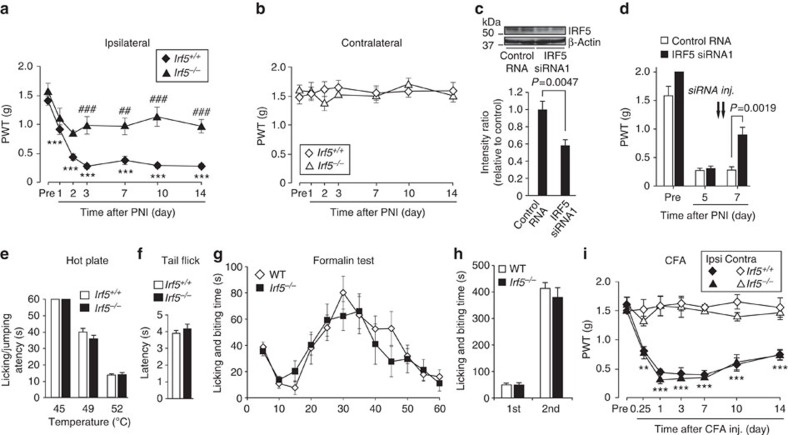
Loss of IRF5 abrogates PNI-induced tactile allodynia without affecting acute pain sensation or inflammatory pain. (**a**,**b**) PWT of *Irf5*^−/−^ and WT littermates (*Irf5*^+/+^) before and after PNI (*n=*6; ***P<0.001 versus Pre; ##*P<*0.01, ###*P<*0.001 versus the ipsilateral side of WT mice). (**c**) Upper: representative western immunoblots of IRF5 in the spinal cords of mice treated with control or IRF8 siRNAs 7 days after PNI. Lower: relative band density ratios of IRF5 (normalized to β-actin) to control RNA (*n=*6). (**d**) Reversal of PNI-induced allodynia by intrathecal administration of IRF5 siRNA (20 pmol) once a day for 2 days (on day 5 and 6 after PNI) in WT mice (*n=*6). (**e**) Hot-plate test of which values represent the latencies for animals to lick their hindpaws or jump (*n=*6). (**f**) Tail-flick test of which values represent the latencies to flick their tail from the heat source (*n=*6). (**g**) Formalin test of which values indicate the duration of nociceptive behaviours (*n=*8). (**h**) Total duration (sec) of nociceptive behaviours for 0–5 min (1st phase) and for 10–60 min (2nd phase) (*n=*8). (**i**) PWT of *Irf5*^+/+^ and *Irf5*^−/−^ mice before and after intraplantar CFA injection (*n=*4, **P<0.01, ***P<0.001 versus Pre). Values are the mean±s.e.m. Full-size blots are shown in Supplementary Fig. 12.

Our results suggest that the IRF8–IRF5 transcriptional network controls the expression of P2X4R in reactive microglia of the spinal dorsal horn after PNI. Microglial P2X4R signals are critical for generating neuropathic pain^12^,^13^,^35^. Therefore, we next examined the functional relevance of IRF5 to tactile allodynia, a hallmark symptom of neuropathic pain characterized by abnormal pain hypersensitivity evoked by innocuous stimuli^10^. Allodynia was assessed by the paw withdrawal threshold (PWT) to mechanical stimulation of the hindpaw. In WT littermates (*Irf5*^*+/+*^), PNI produced a robust and sustained reduction in PWT of the injured side (Fig. 6a). The decrease in PWT on day 1 was comparable between *Irf5*^*−/−*^ and *Irf5*^*+/+*^ mice. After 3 days, PWT in *Irf5*^*−/−*^ mice was not further decreased (Fig. 6a). The behavioural difference between the two genotypes lasted until the final time point tested (day 14, *Irf5*^*+/+*^: 0.275±0.040 g, *Irf5*^*−/−*^: 0.966±0.117 g; Fig. 6a). In contrast, basal mechanical sensitivity or contralateral PWT remained unchanged in *Irf5*^*−/−*^ mice after PNI (Fig. 6a,b). Moreover, tail-flick and paw-flick (hot-plate tests) tests showed that acute physiological pain responses were the same between *Irf5*^*+/+*^ and *Irf5*^*−/−*^ mice (Fig. 6e,f). These results exclude the possibility of congenital defects in pain processing in *Irf5*^*−/−*^ mice and also indicate that the deficiency of IRF5 does not affect general pain sensation. To further test the role of IRF5 in established pain hypersensitivity, we intrathecally administered small interfering RNA (siRNA) targeting IRF5 after PNI. This siRNA markedly suppressed the expression of IRF5 protein and ameliorated the established allodynia (Fig. 6c,d). An additional IRF5 siRNA also produced similar effects on pain behaviour (Supplementary Figs 6a, b and 15). These results indicate that continuous activity of microglial IRF5 in the spinal cord is required for chronic neuropathic allodynia after PNI.

We also assessed behavioural responses in two different inflammatory pain models. Pain responses caused by injecting dilute formalin into the hindpaw (eliciting typical two-phase pattern of behavioural responses^8^) were indistinguishable between the two genotypes (Fig. 6g,h). Furthermore, intraplantar injection of complete Freund’s adjuvant (CFA), an established chronic inflammatory pain model^8^, caused a comparable reduction in PWT in both genotypes (Fig. 6i). In line with this result, expression of spinal IRF5 expression following CFA injection was unchanged (Supplementary Figs 6c and 15). These findings suggest that microglial IRF5 plays a role in the development and maintenance of chronic neuropathic pain without affecting acute physiological pain and local tissue inflammation-evoked pain.

## Discussion

**Figure 7:**
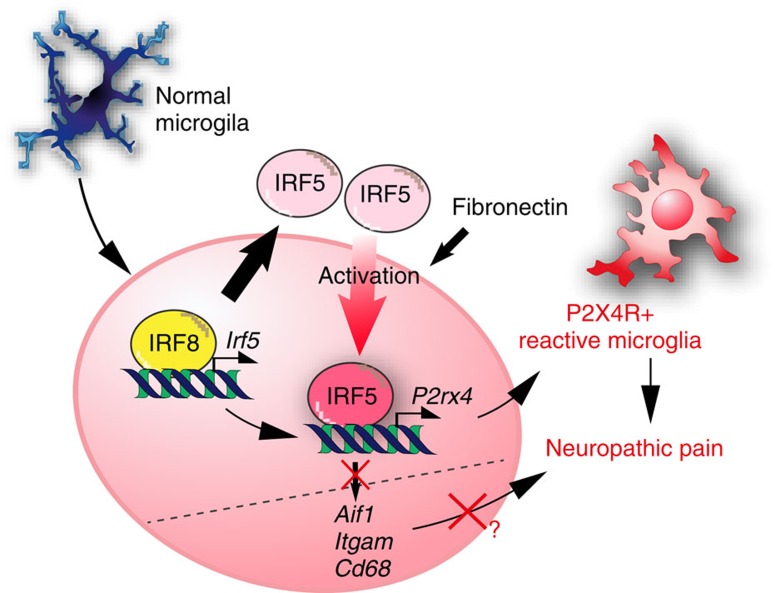
Schematic illustration and working models for determining P2X4R^+^-reactive microglia and neuropathic pain.

In the current study, we report a novel function of the IRF8–IRF5 transcriptional axis in shifting microglia toward the P2X4R^+^ phenotype, by which microglia play a crucial role in the pathogenesis of neuropathic pain (Fig. 7). To our knowledge, this report is the first to identify a microglia-specific transcription network that determines the distinct phenotypes of microglia.

Our detailed analyses reveal that following PNI, IRF5 is increased in spinal microglia in a cell-type-specific manner, depending on IRF8 through its direct binding to the promoter loci of IRF5 and activating the transcription of IRF5. Although the fact that the PNI-induced increased expression of IRF5 in the spinal cord was not completely eliminated in IRF8-deficient mice suggests that IRF8-mediated signals is not the only mechanism underlying upregulation of IRF5, our data strongly suggest that IRF8 acts as a crucial and dominant regulator of IRF5 expression.

Ectopic expression of IRF5 in cultured microglia did not induce any observable signs of microglial activation. Previous reports have shown that TLR signals activate gene expression via the IRF5 pathway in macrophages^17^,^18^,^19^. However, under our experimental conditions, the TLR4 ligand, LPS, did not induce the translocation of microglial IRF5, which may be a result of a lower dependency of IRF5 on the TLR-mediated signalling pathway in microglia. This response corroborates with our findings that microglial gene expressions in response to TLR stimulation (via the TLR3 agonist Poly(I:C), or LPS) were only modestly changed by the knockdown of IRF5 (Supplementary Fig. 7). Previous studies have shown that fibronectin-mediated signals play a crucial role in the upregulation of P2X4R in microglia^26^,^29^. Furthermore, fibronectin stimulation causes IRF5 to cluster on the putative promoter loci of P2X4R. Therefore, activation of IRF5 following fibronectin treatment in this study provided an interesting insight into a previously unknown function of microglial IRF5. We thus propose that IRF5 is an entirely novel transcription activator working at the promoter of P2X4R, thereby inducing *de novo* expression of P2X4R in microglia. The enhanced expression of IRF5 may allow microglia to elicit these responses more quickly and efficiently.

Our results also show that IRF5 deficiency affected IRF8-mediated upregulation of P2X4R, and yet the mechanism underlying this response remains unknown. Post-translational modifications, such as Lys63-linked polyubiquitination, have been considered as an important mechanism to control the localization or function of signalling molecules, including transcription factor^36^. IRF5 harbours a consensus binding site for the ubiquitin ligase tumour necrosis factor receptor (TNFR)-associated factor 6 (TRAF6)^17^,^37^, which is known to regulate Lys63-linked polyubiquitination of its substrates^38^. Furthermore, IRF8 has an enhancing effect on TRAF6-induced ubiquitination^39^. Therefore, in IRF8-transduced microglial cells, IRF5 may be activated via TRAF6-mediated polyubiquitination, resulting in enhanced expression of P2X4R in these cells. Although the molecular machineries of both fibronectin- and IRF8-mediated activation of IRF5 remain to be elucidated, our present data strongly suggest that the expression level of microglial P2X4R is concertedly regulated by IRF5.

Our results demonstrating an IRF8–IRF5–P2X4R axis in microglia may also raise the possibility that a PNI-associated signal that causes upregulation of IRF8 in spinal microglia results in inducing IRF5-mediated P2X4R expression. Although such signal remains to be identified, to determine whether the signal itself has a cooperative effect on IRF5-mediated P2X4R expression in microglia would be now a new important open question for future study.

Microglia have often been designed as M1 or M2 macrophage-like^40^,^41^,^42^. Previous studies have shown that IRF5 is involved in the polarization of M1 macrophages, which exhibit progressive expressions of inflammatory molecules, such as pro-inflammatory cytokines^18^. However, gene expressions in the spinal cord of IRF5-deficient mice in the present study did not follow the expected patterns. Deficiency of IRF5 did not lead to a global defect in the expression of inflammatory components (for example, *Il1b*, *Il6* and *Tnfa*), but affected the P2X4R–BDNF axis in the spinal cord in a restricted manner following PNI, though whether stimulating P2X4Rs is required for BDNF expression in microglia after PNI remains to be determined^13^,^15^,^16^. Our data indicate that reactive spinal microglia after PNI cannot be classified into either reactive modality, but rather exhibit a neuropathic pain-specific signature. In addition, the function of IRF5 was found to be cell type- and location-dependent, which is consistent with previous reports^19^,^43^.

Our previous study identified IRF8 as a key transcription regulator of microglia for its transformation into a reactive state and its involvement in the pathogenesis of neuropathic pain after PNI^8^. In the CNS parenchyma of *Irf8*^*−/−*^ mice, microglial cells exhibit an abnormal morphology without fine processes^44^,^45^, by which microglia sense pathological alterations or disturbances to maintain homeostasis, suggesting that excessive repression of microglial IRF8 may inhibit the physiological functions of these cells. Therefore, defining transcriptional control mechanisms that participate or occur downstream of IRF8 and regulate specialized microglial genes (for example, P2X4R) involved in neuropathic pain may be more favourable for treating chronic pain conditions. Therefore, IRF5 may be a potential therapeutic target because our data reveal that *Irf5*^−/−^ mice exhibit less allodynic behaviour, with microglial morphology comparable to WT mice, which also suggests that PNI-induced morphological alteration of spinal microglia may not sufficiently contribute to the pathogenesis of neuropathic pain. Furthermore, our results demonstrating that suppressed spinal IRF5 by siRNA reverses pain hypersensitivity indicate that ongoing activity of microglial IRF5 is required for the development and maintenance of neuropathic pain, possibly through the P2X4R pathway.

In conclusion, this study provides a new insight into the mechanisms underlying the phenotypic shifts of microglia in response to injury or disease in the nervous system. Although the functions of microglial IRF5 in other CNS diseases is unknown, our results suggest a key transcriptional network that forms ‘neuropathic pain-driving microglia’, which can provide a potential therapeutic target.

## Methods

### Animals

Male IRF5-deficient mice (Irf5^−/−^)^17^, IRF8-deficient mice (Irf8^−/−^)^46^ and their wild-type littermates, and C57BL/6 mice (Clea, Japan) were used. All mice used were aged 9–12 weeks at the start of each experiment, and were housed individually and in groups of two or three per cage at a temperature of 22±1 °C with a 12-h light–dark cycle, and were fed food and water *ad libitum*. All experimental procedures were performed under the guidelines of Kyushu University.

### Microglial culture

Neonatal mouse brains were minced with a razor blade, and trypsinized in phosphate-buffered saline (PBS) containing 0.25% trypsin and 0.05% DNase at 37 °C. After adding fetal bovine serum, the dissociated cells were filtered and maintained in flasks for 10–16 days in DMEM containing 10% fetal bovine serum. Immediately before experiments, microglia were collected by a gentle shake as the floating cells over the mixed glial culture^8^. The microglia were transferred to dishes for subsequent experiments.

### Peripheral nerve injury

We used the spinal nerve injury model^47^ with some modifications^8^. Under isoflurane (2%) anaesthesia, a small incision at L3–S1 was made. Paraspinal muscle and fat were removed from the L5 transverse process, and the part of this transverse process was removed to expose the parallel-lying L3 and L4 spinal nerves, and then the L4 nerve was carefully isolated and cut. The wound and the surrounding skin were sutured with 5-0 silk.

### Microarray analysis

Extraction of total RNA from the L3–L4 spinal cord using TRIsure (Bioline) and purification using RNeasy mini plus kits (Qiagen) were performed^48^. Total RNA was converted to biotin-labelled cRNA, which was hybridized to the Mouse WG-6 V2.0 BeadChip (Illumina). Multiple gene expression in the spinal cord with PNI was analysed according to BeadStudio Gene Expression Module User Guide (Illumina).

### Quantitative real-time PCR

Mice were deeply anaesthetized with pentobarbital, perfused transcardially with PBS and the L3–L4 spinal cord was removed immediately. The tissues were vertically separated by median, and hemisections of the spinal cord were subjected to total RNA extraction using Trisure (Bioline) according to the protocol of the manufacturer and purified with RNeasy mini plus kit (Qiagen, Valencia, CA, USA). Extraction of total RNA from primary cultured microglia or the microglial cell line BV-2 (provided by Dr Knut Biber, University of Freiburg) was also performed using TRIsure. The amount of total RNA was quantified by measuring OD260 using a Nanodrop spectrophotometer (Nanodrop, Wilmington, DE, USA). For reverse transcription, 150 ng of total RNA was transferred to the reaction with Prime Script reverse transcriptase (Takara). Quantitative PCR was performed with FastStart Essential DNA Probes Master (Roche) using a LightCycler 96, a LightCycler 480 system (Roche) or with Premix Ex Taq (Takara) using a 7500 real-time PCR system (Applied Biosystems, Foster City, CA, USA). Expression levels were normalized to the values for 18s ribosomal RNA. The sequences of TaqMan primer pairs and probe are described below.

IRF5: 5′-CCTCAGCCGTACAAGATCTACGA-3′ (forward), 5′-GTAGCATTCTCTGGAGCTCTTCCT-3′ (reverse), 5′-FAM-CCAACGGCCCTGCTCCCACA-TAMRA-3′ (probe)

IRF8: 5′-GGATATGCCGCCTATGACACA-3′ (forward), 5′-CATCCGGCCCATACAACTTAG-3′ (reverse), 5′-FAM-CCATTCAGCTTTCTCCCAGATGGTCATC-TAMRA-3′ (probe)

Iba1(Aif1): 5′-GATTTGCAGGGAGGAAAAGCT-3′ (forward), 5′-AACCCCAAGTTTCTCCAGCAT-3′ (reverse), 5′-FAM-CAGGAAGAGAGGCTGGAGGGGATCAA-TAMRA-3′ (probe)

CD68: 5′-CTGCTGTGGAAATGCAAGCATA-3′ (forward), 5′-CCCGAAGTGTCCCTTGTCA-3′ (reverse), 5′-FAM-TCTCTCTAAGGCTACAGGCTGCTCAGCTGC-TAMRA-3′ (probe)

TLR2: 5′-CCCTTCTCCTGTTGATCTTGCT-3′ (forward), 5′-CGCCCACATCATTCTCAGGTA-3′ (reverse), 5′-FAM-CTGTGCCACCATTTCCACGGACTG -TAMRA-3′ (probe)

TLR4: 5′-AAACTTGCCTTCAAAACCTGGC-3′ (forward), 5′-ACCTGAACTCATCAATGGTCACATC-3′ (reverse), 5′-FAM-CACGTCCATCGGTTGATCTTGGGAGAA-TAMRA-3′ (probe)

CX3CR1: 5′-TCACCGTCATCAGCATCGA-3′ (forward), 5′-CTGCACTGTCCGGTTGTTCA-3′ (reverse),

5′-FAM-ATCGTCCTGGCCGCCAACTCC-TAMRA-3′ (probe)

P2X4R: 5′-ACAACGTGTCTCCTGGCTACAAT-3′ (forward), 5′-GTCAAACTTGCCAGCCTTTCC-3′ (reverse), 5′-FAM-CAATGAGCAACGCACACTCACCAAGG-TAMRA-3′ (probe)

P2X7R: 5′-TGCAGCTGGAACGATGTCTT-3′ (forward), 5′-CCAAAGCAAAGCTCTAATGTAGGAA-3′ (reverse), 5′-FAM-TATGAGACAAACAAAGTCACCCGGATCCA-TAMRA-3′ (probe)

P2Y12R: 5′-TGAAGACCACCAGGCCATTT-3′ (forward), 5′-AGGCCCAGATGACAACAGAAA-3′ (reverse), 5′-FAM-AAACGTCCAGCCCCAGCAATCTCTTG-TAMRA-3′ (probe)

IL-1β: 5′-GAAAGACGGCACACCCACC-3′ (forward), 5′-AGACAAACCGCTTTTCCATCTTC-3′ (reverse), 5′-FAM-TGCAGCTGGAGAGTGTGGATCCCAA-TAMRA-3′ (probe)

IL-6: 5′-GGGACTGATGCTGGTGACAA-3′ (forward), 5′-TGCCATTGCACAACTCTTTTCT-3′ (reverse), 5′-FAM-TCACAGAGGATACCACTCCCAACAGACCTG-TAMRA-3′ (probe)

BDNF: 5′-GCCCAACGAAGAAAACCATAAG-3′ (forward), 5′-TGTTTGCGGCATCCAGGTA-3′ (reverse), 5′-FAM-CACTTCCCGGGTGATGCTCAGCA-TAMRA-3′ (probe)

Cathepsin S (Ctss): 5′-TACATTCAGCTCCCGTTTGGT-3′ (forward), 5′-TCGTCATAGACACCGCTTTTGT-3′ (reverse), 5′-FAM-TCGACGCCAGCCATTCCTCCTTCT-TAMRA-3′ (probe), as well as the primers and probe for 18s were obtained from Applied Biosystems.

### Western blotting

Cultured microglial cells were lysed in lysis buffer (50 mM Tris–HCl (pH 7.4), 150 mM NaCl, 1% NP-40, 0.1% SDS, 0.5% DOC, protease inhibitors cocktail) and mixed with Laemmli sample buffer. For the mouse spinal cord homogenates, mice were deeply anaesthetized with pentobarbital, perfused transcardially with PBS, and the L3–L4 spinal cord was removed immediately. The tissues were vertically separated by median, and then hemisections of the spinal cord were homogenized in homogenization buffer (20 mM Tris–HCl (pH 7.4), 2 mM EDTA, 0.5 mM EGTA, 0.32 M sucrose, protease and phosphatase inhibitor cocktails) and centrifuged to remove cell debris. The supernatant was transferred to a new tube, mixed with Laemmli sample buffer and boiled at 95 °C. For isolation of nuclear and cytoplasmic protein, BV2 cells were lysed with buffer A (10 mM HEPES (pH 7.5), 10 mM KCl, 1.5 mM MgCl_2_, 0.1 mM EGTA, 0.05% NP-40, 1.0 mM DTT, protease inhibitor cocktail), and then nuclei were recovered by centrifugation. The supernatant, which contained the cytoplasmic and membrane protein fractions, was collected for western blot analysis. Nuclear proteins were extracted by resuspending the nuclei pellet in buffer B (20 mM HEPES (pH 7.5), 25% (*v*/*v*) glycerol, 0.42 M NaCl, 1.0 mM EGTA, 1.0 mM EDTA, 1.0 mM DTT, protease inhibitor cocktail). After centrifugation, the supernatant that contained the nuclear protein fraction was collected. Aliquots (0.5–20 μg) were subjected to a 10% polyacrylamide gel electrophoresis, and proteins were transferred electrophoretically to PVDF membranes. After blocking with blocking one (Nacalai tesque), the membranes were incubated with anti-IRF5 rabbit polyclonal antibody (1:500, Abcam), anti-IRF8 goat polyclonal antibody (1:500, Santa Cruz Biotechnology), anti-P2X4R rabbit polyclonal antibody (1:1,000, Alomone), anti-β-actin mouse monoclonal antibody (1:2,000, Sigma), anti-α-tublin polyclonal antibody (1:1,000, Abcam), anti-lamin B polyclonal antibody (1:1,000, Santa Cruz) and then incubated with HRP-conjugated secondary antibody (1:1,000, GE Healthcare). The blots were detected using a chemiluminescence method (Chemi-Lumi One; Nacalai tesque) and exposed to films. Bands were quantified using the software NIH Image J 1.36, and the relative values of each protein were normalized by the values of the protein levels of β-actin, α-tublin or lamin B for the loading control.

### ChIP-qPCR assay

ChIP assays were performed with SimpleChIP Enzymatic Chromatin IP Kit (Cell Signaling Technology Japan, Tokyo, Japan) with anti-IRF1 (Santa Cruz Biotechnology), anti-IRF3(Santa Cruz Biotechnology), anti-IRF5 (Abcam), anti-IRF7 (Santa Cruz Biotechnology), anti-IRF8 (Santa Cruz Biotechnology), anti-IRF9 (Santa Cruz Biotechnology), normal goat IgG (Abcam) and normal rabbit IgG (Cell Signaling Technology) antibodies. ChIP signals were quantified by quantitative PCR analysis with a 7500 real-time PCR system (Applied Biosystems). Values obtained for immunoprecipitated samples (percent (%) input DNA) were normalized to values for respective normal IgG. The specific primer pairs for the *Irf5* promoter region and *P2rx4* promoter region, respectively, are described below.

Region 1: 5′-ATTTCTCAGGCCCTGTCTAAAGTG-3′ (forward),

5′-GGCACAGAGAGAGTTAGAGGAAGA-3′ (reverse)

Region 2: 5′-TATGGAGTCTTTCTGCACCCTGT-3′ (forward),

5′-TTCCAAGAACGAAGAGTCCCCTA-3′ (reverse)

ISRE-1: 5′-GCTGGCTCGTTTCAAGAATATT-3′ (forward),

5′-CGTACCCTGTAGCCGTCTATT-3′ (reverse)

ISRE-2: 5′-TCTACAGCCTGAAAGTCTATCATTG-3′ (forward),

5′-AAGGAATCTGAGAGGTACACACTG-3′ (reverse)

ISRE-3: 5′-GATAGGGAGAGGCTCGTTCA-3′ (forward),

5′-TAAAAGCTCGGGACCTGGAA-3′ (reverse)

ISRE-4: 5′-TACTGACCTGCCTCTTTTAAGGACA-3′ (forward),

5′-CGGAAAGAACTTTGAACCTTGAG-3′ (reverse)

### Immunohistochemistry

Mice were deeply anaesthetized by pentobarbital and perfused transcardially with PBS followed by ice-cold 4% paraformaldehyde/PBS. The L4 segment of the lumbar spinal cord were removed, post-fixed in the same fixative and placed in 30% sucrose solution for 24 h at 4 °C. Transverse L4 spinal cord sections (30 μm) were incubated for 48 h at 4 °C with primary antibody. Identification of cell types was performed using the following markers: microglia, Cd11b (1:1,000, Serotec), Iba1 (ionized calcium-binding adapter molecule-1, 1:2,000, Wako) and Cd68 (1:1,000, Serotec); astrocytes, GFAP (1:500, Chemicon); neurons, NeuN (Neuronal Nuclei, 1:200, Chemicon). Spinal sections were incubated with secondary antibodies conjugated to Alexa Fluor 488 or 546 (1:1,000, Molecular Probes) and mounted in VECTASHIELD with or without DAPI (Vector Laboratories). Three to five sections from the L4 spinal cord segments of each mouse were randomly selected and analysed using an LSM510 Imaging System (Carl Zeiss, Japan).

### Lentiviral transduction

The lentiviral CS2 vectors (RIKEN) encoding mouse IRF8–GFP, IRF8(K79E)–GFP, IRF5-GFP or GFP under the control of the human EF-1α promoter, or encoding IRF5 or control shRNA sequences expressed under the control of H1 promoter upstream of the EF-1α promoter–IRF8-GFP or GFP expression cassette were used. Each vector with pCAG-HIVgp (packaging plasmid; RIKEN), and pCMV-VSV-G-RSV-Rev (RIKEN) was cotransfected into HEK293T cells. After mixing with PEG, viral particles and 8 μg ml^−1^ polybrene were added onto primary cultured microglia (1.2 × 10^5^ cells per well) or microglial BV2 cells (1.0 × 10^4^ cells per well) plated on 24-well plates^49^. After a 12-hour treatment with the lentivirus, cultured medium was changed to conditioned medium prepared from the mixed glial culture, and microglia were further cultured for 60 h. For the gene expression experiments, the transduced microglia were subjected to total RNA extraction as described above.

### Luciferase assay

To construct the Irf5 gene reporter plasmid, the 928-bp fragment from the Irf5 gene promoter was amplified by PCR from genomic DNA. The PCR products were subcloned into the region upstream of a luciferase expression cassette of the CS2 vector. Subsequently, viral particles carrying this cDNA were produced as stated above. Normal or IRF8-transduced BV2 cells were transduced with them. Then, the cells were plated, followed by lysing with Glo lysis buffer and the supernatant was assayed for luciferase activity with the Bright-Glo Luciferase Assay System (Promega).

### Intrathecal injection of siRNA

Under isoflurane (2%) anaesthesia, mice were implanted with a 32-gauge intrathecal catheter (ReCathCo, Allison Park, PA, USA) through the atlanto-occipital region and in the lumbar enlargement (close to L3–L4 segments) of the spinal cord. After 7 days of recovery, the catheter placement was verified by the observation of transient hindpaw paralysis induced by intrathecal injection of lidocaine (2%, 1.5 μl). Animals that failed to show any paralysis were not used in experiments. Intrathecal injection of IRF5 siRNA or scrambled control siRNA (10 pmol per 2.5 μl) was followed by infusion of 3 μl of PBS. Sequences of the IRF5 siRNAs and the non-targeting scramble siRNA used in this study were as follows.

IRF5 siRNA-1 sense sequence: 5′-GCAGUUUAAAGAGCUUCAUUU-3′

IRF5 siRNA-1 antisense sequence: 5′-AUGAAGCUCUUUAAACUGCUU-3′.

IRF5 siRNA-2 sense sequence: 5′-GCCUAGAGCAGUUUCUCAAUU-3′.

IRF5 siRNA-2 antisense sequence: 5′-UUGAGAAACUGCUCUAGGCUU-3′.

Scramble siRNA sense sequence: 5′-GUUAGAAAGGGCAGAUAAAUU-3′.

Scramble siRNA antisense sequence: 5′-UUUAUCUGCCCUUUCUAACUU-3′.

### Behavioural studies

To assess mechanical sensitivity, mice were placed individually in an opaque plastic cylinder, which was placed on a wire mesh and habituated for 1 h to allow acclimatization to the new environment. After that, calibrated von Frey filaments (0.02–2.0 g, North Coast Medical) were applied to the plantar surfaces of the hindpaws of mice with or without PNI, or peripheral inflammation caused by intraplantar injection of CFA (0.01 mg per 20 μl, Sigma), and the 50% PWT was determined using the up–down method^50^. In a hot-plate test, mice were placed on a metal surface maintained at 45, 49 or 52 °C within a 25-cm-high Plexiglas box (25 cm  ×  2). The latency to either lick or shake the hindpaw or jump was measured as a nocifensive end point. Noxious heat-evoked tail flick responses were detected by the application of radiant heat (Ugo Basile, Italy) to the tail. The intensity of the heat stimulus was adjusted to 50 V, and the latency of the tail withdrawal response (seconds) was measured. In the tests of formalin-induced pain, mice were housed in individual box and allowed to habituate to the testing environment for 15 min. Then, mice were injected intraplantarly with formalin (5%, 20 μl), and then the duration of the licking and biting responses to the injected hindpaw was recorded at 5 min intervals for 60 min after the injection. All behavioural assessments were performed by an observer blind to experimental condition.

### Statistical analysis

Statistical significance of differences was determined using the Student’s *t* test (Figs 3k and 6c,d), two-way ANOVA with *post hoc* Bonferroni test (Figs 1c,e and 6a), one-way ANOVA with a *post hoc* Dunnett’s multiple comparison test (Figs 3a and 6a,i), Bonferroni multiple comparison test (Figs 2c,e, 3b,c and 4b–f), or Tukey’s multiple comparison test (Figs 2b, 3f–h and 5a,d) using GraphPad Prism 5.04 software. Differences were considered significant at *P<*0.05.

## Author contributions

T.M. designed and performed most of the experiments, analysed the data, and wrote the manuscript. S.I. performed ChIP-qPCR assays and *in vitro* experiments. R.Y., H.T.-S. and A.N. assisted with the experiments. T.W.M. kindly provided *Irf5*^−/−^ mice. T.T. provided critical materials including *Irf8*^−/−^ mice and advice on data interpretation. M.T. conceived the study, supervised the overall project, designed the experiments and wrote the manuscript. K.I. supervised the overall project and wrote the manuscript.

## Additional information

**How to cite this article:** Masuda, T. *et al.* Transcription factor IRF5 drives P2X4R^+^-reactive microglia gating neuropathic pain. *Nat. Commun.* 5:3771 doi: 10.1038/ncomms4771 (2014).

## Supplementary Material

Supplementary Information Supplementary Figures 1-15
