# Supplementary material for: Transcription factor IRF5 drives P2X4R+-reactive microglia gating neuropathic pain
Source: Nat Commun. 2014 May 13;5:3771. doi: 10.1038/ncomms4771 (PMC4024744; doi:10.1038/ncomms4771)
Supplement: Supplementary Information — Supplementary Figures 1-15 [file ncomms4771-s1.pdf]

Supplementary Information

Transcription factor IRF5 drives P2X4R$^+$ reactive microglia gating neuropathic pain

Takahiro Masuda, Shosuke Iwamoto, Ryohei Yoshinaga, Hidetoshi Tozaki-Saitoh, Akira Nishiyama, Tak W. Mak, Tomohiko Tamura, Makoto Tsuda, Kazuhide Inoue

Supplementary Figures S1-S15 and reference

a

Microarray data

Gene	Ipsilateral (SEM)	Contralateral (SEM)	Ipsi/Contra	Student' s *t* test
Irf1	129.5 (6.3)	125.2 (6.5)	1.03	*P*>0.05
Irf2	1363.4 (77.4)	1554.1 (169.2)	0.88	*P*>0.05
Irf3	178.1 (3.0)	189.6 (6.8)	0.94	*P*>0.05
Irf5	192.54 (7.73)	133.84 (3.64)	1.44	*P<0.001*
Irf9	197.8 (12.0)	145.4 (5.4)	1.36	*P*=0.016

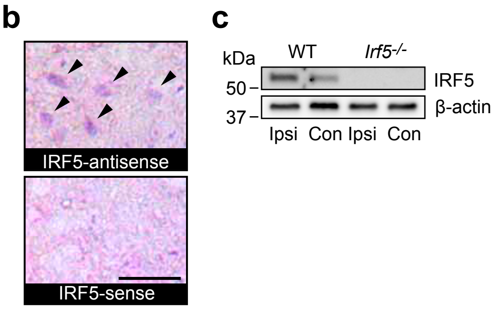

Supplementary Figure 1. Microglia-specific IRF5 upregulation following PNI. **(a)** Expression of IRF family transcription factors in the spinal cord 7 days after PNI by three or four independent DNA microarray analyses. The data of three members (IRF4, IRF6 and IRF7) were excluded from the statistics because of a detection p-value of greater than 0.01 (n=3 or 4; Student' s *t* test). IRF8 has already been identified as a upregulated gene in the ipsilateral spinal cord after PNI[8]. **(b)** *In situ* hybridization (ISH) analysis of *Irf5* mRNA in the spinal dorsal horn of WT mice 7 days after PNI. Arrowheads indicate *Irf5* mRNA signals. (Scale bar, 30 μm) **(c)** Western blot analysis of IRF5 and β-actin proteins in the spinal cords of WT and *Irf5*$^{-/-}$ mice 3 days after PNI. Ipsi, ipsilateral; Con, contralateral. Full-size blots are shown in Supplementary Fig. 13.

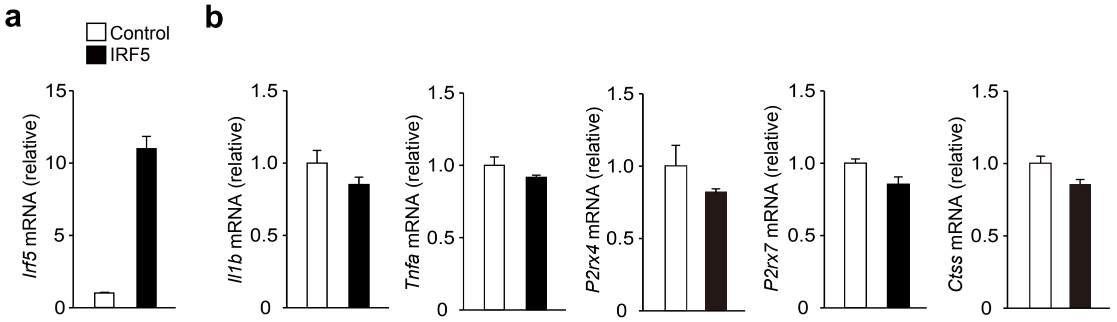

Supplementary Figure 2. Ectopic expression of IRF5 itself did not activate transcription of genes in microglia.

Real-time PCR analysis of mRNAs of **(a)** *Irf5* and **(b)** microglial genes in BV2 cells transduced with IRF5-GFP or GFP alone (control) (n=3). Values are means ± s.e.m..

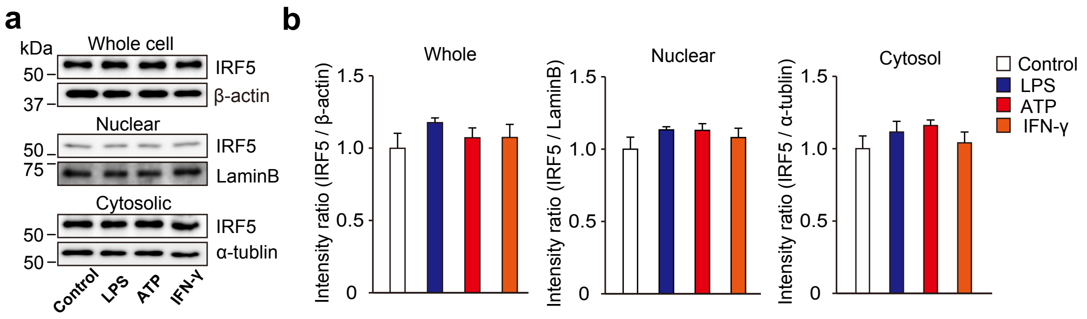

Supplementary Figure 3. LPS, ATP or IFN-γ did not induce translocation of IRF5 into the nucleus in microglia.

(a) Expressional distribution analysis of IRF5 protein in microglial BV2 cells treated with LPS (100 ng/ml), ATP (50 μM) and IFN-γ (100 U/ml) for 4 h. **(b)** A histogram of the relative band intensity ratio of IRF5 (normalised to β-actin, lamin B, or α-tublin) to control cells (n=4). Values are means ± s.e.m.. Full-size blots are shown in Supplementary Fig. 14.

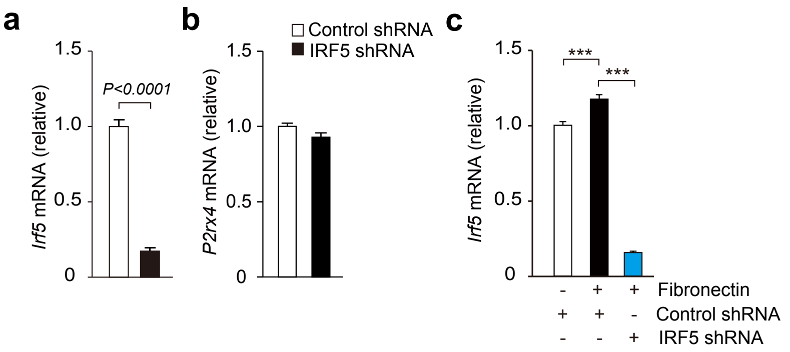

Supplementary Figure 4. Knockdown of microglial IRF5 expression by lentiviral-mediated transduction of IRF5 shRNA.

(a, b) Real-time PCR analysis of *Irf5* **(a)** and *P2rx4* **(b)** mRNA in BV2 cells transduced with a lentiviral vector encoding either IRF5 shRNA or control shRNA. Values represent the relative ratio of *Irf5* or *P2rx4* mRNA (normalised to *18s* mRNA) to the cells with control shRNA (n= 6, Student' s *t* test). **(c)** Expression of *Irf5* mRNA in IRF5 or control shRNA-transduced BV2 cells treated with fibronectin (n=6, ****P<0.001,* one-way ANOVA with Bonferroni's multiple comparison test). Values are means ± s.e.m..

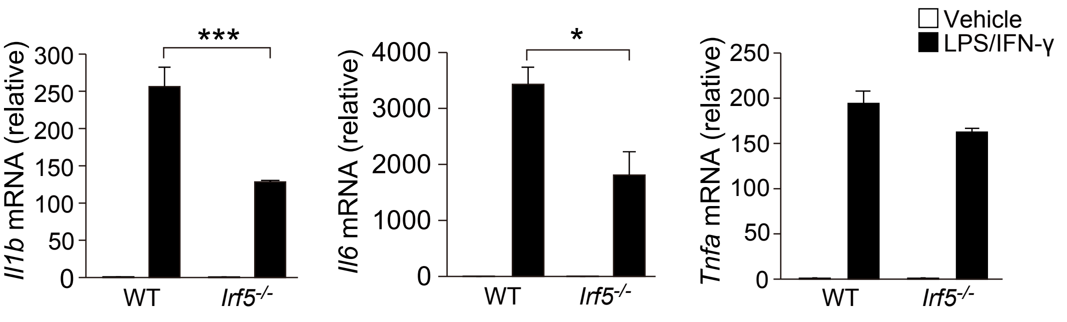

Supplementary Figure 5. Impaired production of proinflammatory cytokines in *Irf5*$^{-/-}$ macrophages.

Real-time PCR analysis of mRNAs of *Il1b*, *Il6* and *Tnfa* in WT and *Irf5*$^{-/-}$ peritoneal macrophages treated with or without LPS (10 ng/μl) / IFN-γ (100 units/ml) for 4h. Values represent the relative ratio of *Il1b*, *Il6* or *Tnfa* mRNA (normalised to the value for *18s* mRNA] to WT control cells (vehicle) (n =4, **P<0.05*, ****P<0.001*, one-way ANOVA with Tukey's multiple comparison test). Values are means ± s.e.m..

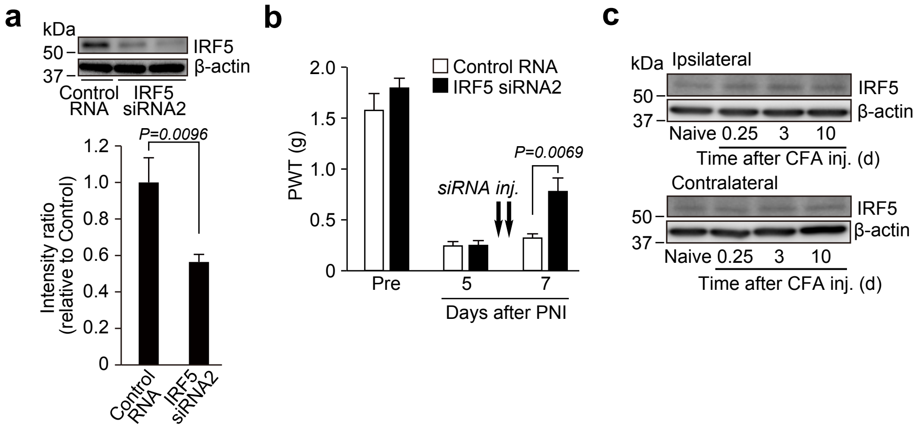

Supplementary Figure 6. PNI-induced ongoing IRF5 expression in the spinal cord is required for tactile allodynia.

(a) Upper, representative immunoblots of IRF5 and β-actin protein in homogenates obtained from the spinal cord of mice treated with control and IRF5 siRNA-2 on day 7 post-PNI. Lower, a histogram of the relative band density ratio of IRF5 (normalised to β-actin) to the control RNA-treated mice (n=6, Student' s *t* test). **(b)** Reversal of PNI-induced tactile allodynia by intrathecal administration of IRF5 siRNA-2 (20 pmol) once a day for 2 days (5 and 6 days post-PNI) in WT mice (n=7, Student' s *t* test). **(c)** Representative western blot analysis of IRF5 protein (of three experiments) in the ipsilateral and contralateral spinal cords of WT mice before (Naive) and after intraplantar CFA injection. Values are the mean ± s.e.m.. Full-size blots are shown in Supplementary Fig. 15.

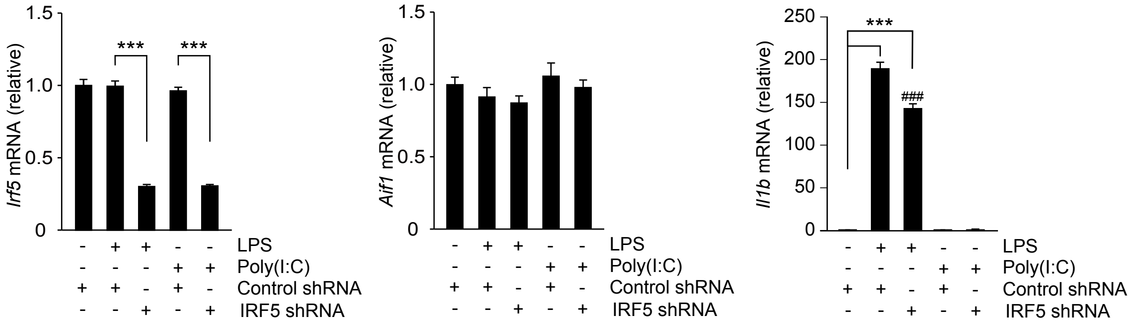

Supplementary Figure 7. Poly(I:C)- or LPS-induced gene expressions in micorglia are only modestly changed by knockdown of IRF5.

Real-time PCR analysis of mRNAs of *Irf5*, *Aif1 and Il1b* in control or *Irf5* shRNA-transduced BV2 cells 6 h after treatment of Poly(I:C) (50 µg/mL) or LPS (100 ng/mL). Values represent the relative ratio of mRNA (normalised to the value for 18s mRNA] to control shRNA-transduced cells (n =6; ****P < 0.001*,###*P < 0.001 vs.* Control shRNA (LPS), one-way ANOVA with Tukey's multiple comparison test). Values are means ± s.e.m..

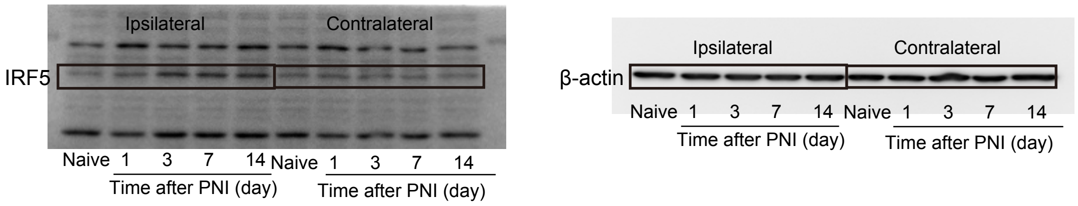

Supplementary Figure 8. Unedited full blots of Figure 1d.

Figure 2a

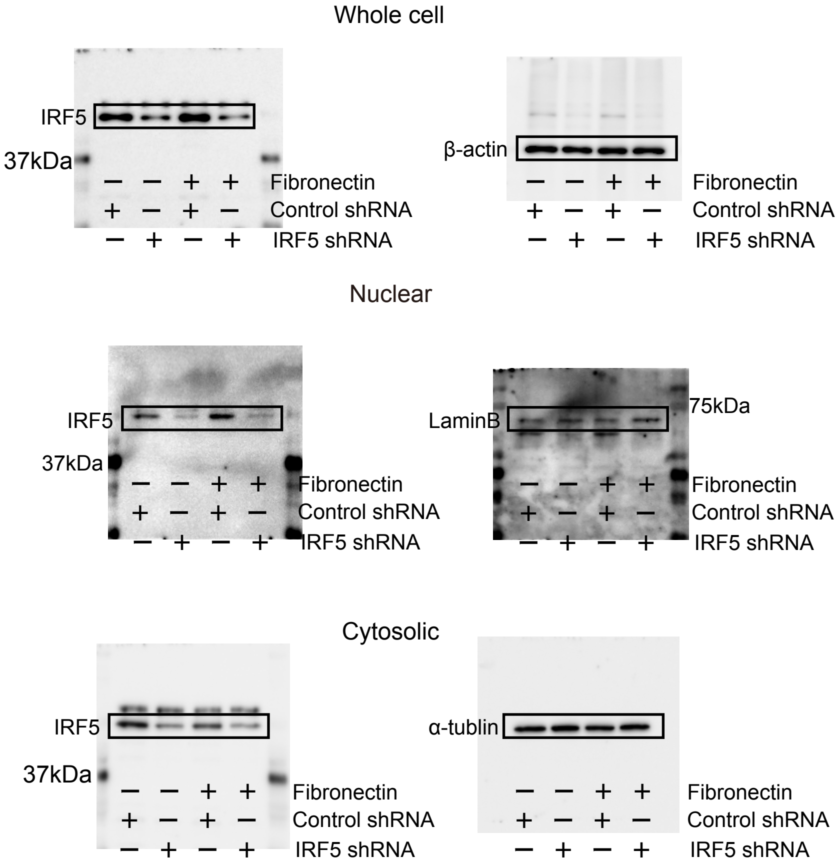

Figure 2d

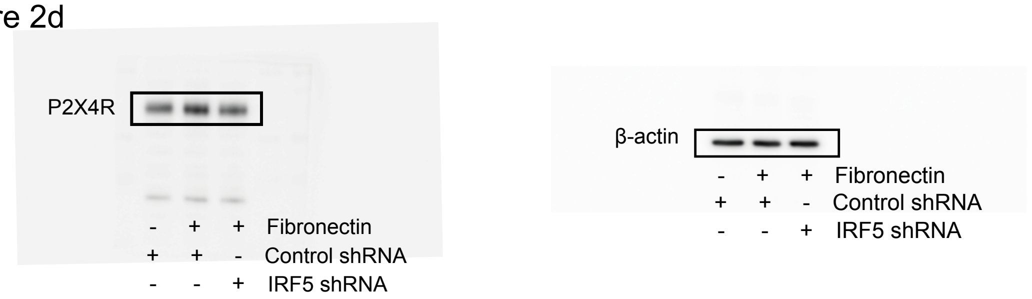

Supplementary Figure 9. Unedited full blots of Figure 2.

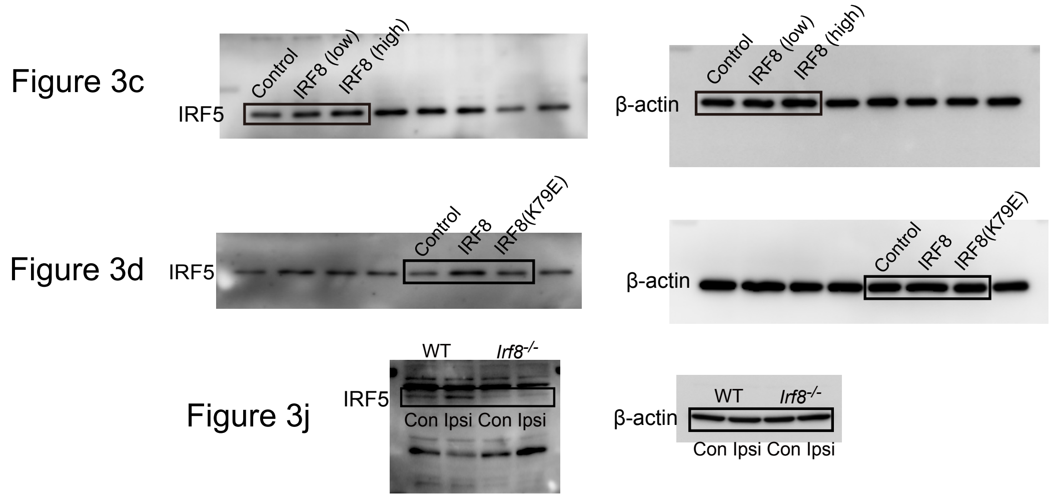

Supplementary Figure 10. Unedited full blots of Figure 3.

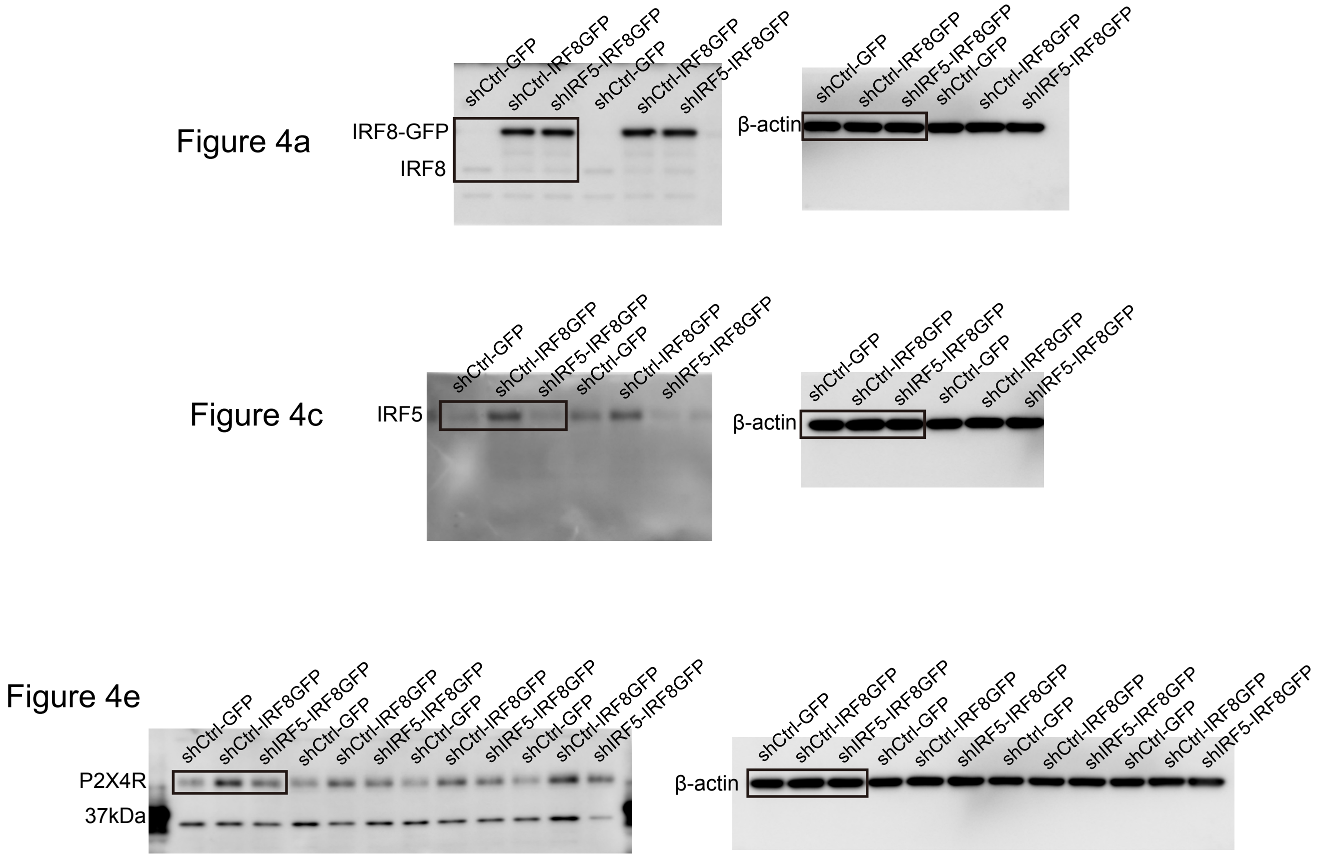

Supplementary Figure 11. Unedited full blots of Figure 4.

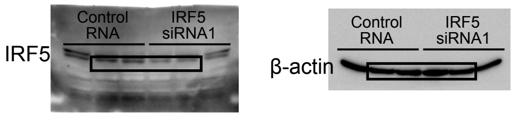

Supplementary Figure 12. Unedited full blots of Figure 6c.

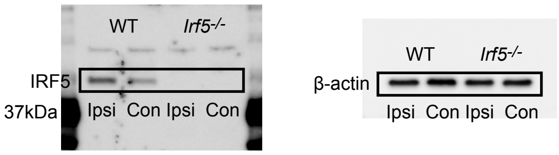

Supplementary Figure 13. Unedited full blots of Supplementary Figure 1c.

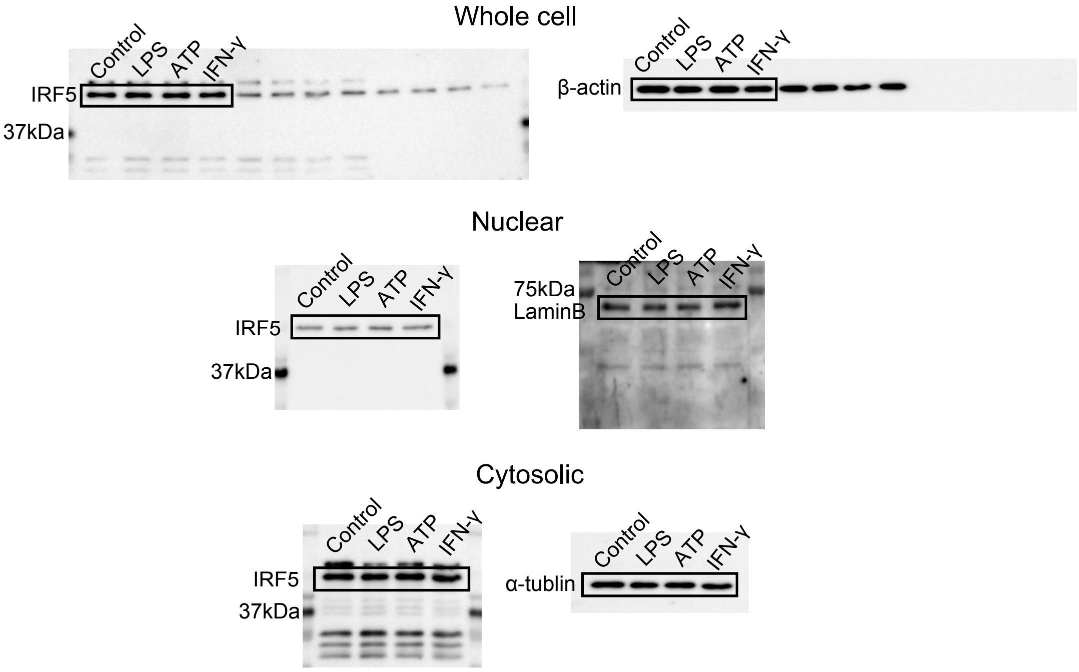

Supplementary Figure 14. Unedited full blots of Figure 3a.

Supplementary Figure 6a

Supplementary Figure 6c

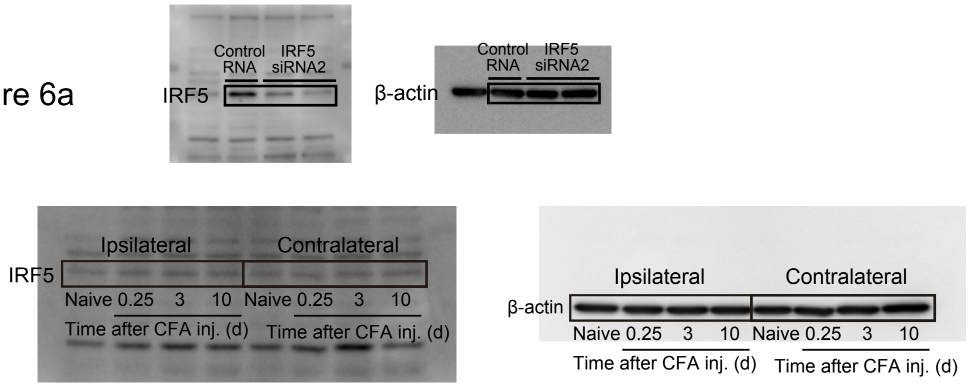

Supplementary Figure 15. Unedited full blots of Supplementary Figure 6.

Supplementary Reference

1. Masuda T, *et al.* IRF8 is a critical transcription factor for transforming microglia into a reactive phenotype. *Cell Rep* **1**, 334-340 (2012).
